# Enhancing the resolution of microseismicity through dense array monitoring in complex extensional settings

**DOI:** 10.1038/s41598-026-35586-3

**Published:** 2026-01-17

**Authors:** Francesco Scotto di Uccio, Titouan Muzellec, Antonio Scala, Grazia De Landro, Giovanni Camanni, Francesco Carotenuto, Luca Elia, Matteo Picozzi, Aldo Zollo, Claudio Strumia, Gregory C. Beroza, Gaetano Festa

**Affiliations:** 1 Department of Physics ‘Ettore Pancini’, Università di Napoli Federico II, Napoli, 80126 Italy; 2 Department of Meteorology and Geophysics, University of Vienna, Vienna, 1090 Austria; 3 Istituto Nazionale di Geofisica e Vulcanologia (INGV), Sezione Osservatorio Vesuviano, Napoli, Italy; 4 Department of Chemical and Geological Sciences, Università degli Studi di Modena e Reggio Emilia, Modena, 41125 Italy; 5 National Institute of Oceanography and Applied Geophysics – OGS, Trieste, Italy; 6 Department of Geophysics, Stanford University, Stanford, CA 94305 USA; 7 Istituto Nazionale di Geofisica e Vulcanologia (INGV), Sezione Osservatorio Nazionale Terremoti, Roma, Italy

**Keywords:** Geophysics, Seismology

## Abstract

Characterizing geometry and mechanics of structures hosting moderate-to-large earthquakes is essential for seismic hazard assessment, yet remains challenging in extensional environments, where fault systems include multiple segments and bends. In this study, we demonstrate how a short-term array deployment can provide critical insights into seismicity patterns and fault geometry in Southern Apennines, Italy.We integrated data recorded by arrays during a one-year experiment with machine learning methodologies, producing a seismic catalog that enhances the manual catalog for the same period by nearly an order of magnitude, lowering completeness magnitude by one unit. Approximately 65% of the detected events can be accurately relocated, with median uncertainties of ~ 100 m, comparable to those of long-term catalogs. Our results reveal consistent seismicity properties down to decametric earthquake size, with hypocenters and b-value mirroring those from the previous decade. We distinguish a shallow, diffuse seismicity, likely influenced by hydrological loading, from deeper clusters, mostly rupturing patches a few-hundred meters across. Beyond asperity-scale complexity, seismicity follows the boundaries of tomographic anomalies, delineating a 50–60 km-long curving fault, featuring a right-stepping jog several kilometers wide. Dynamic simulations suggest that ruptures nucleating on this fault could propagate through these complexities, potentially generating earthquakes up to magnitude 7.0.

**Supplementary Information:**

The online version contains supplementary material available at 10.1038/s41598-026-35586-3.

## Introduction

Detailed information on fault geometry and mechanical conditions provides crucial information that can reduce epistemic uncertainty in ground-motion modeling and seismic hazard assessment^1^. However, anticipating the geometry and even the presence of seismogenic faults presents a challenge, as demonstrated by many recent large earthquakes that occurred on previously unknown faults, such as those responsible for the 2019 M 7.1 Ridgecrest earthquake in California^2^. This issue is even more challenging in complex fault systems that comprise subparallel, segmented synthetic and antithetic structures and interact mechanically over diverse space and time scales^3,4^, where achieving the resolution required to image fault structures typically demands long-term earthquake recordings.

In the actively extending Apennines chain, even moderate-size events with magnitudes ranging from 6 to 6.5, that rupture 10 to 30 km-length faults, may lead to extensive casualties and building damage, as illustrated by the 2009 L’Aquila earthquake^5^ and the 2016 Amatrice-Norcia sequence^6^. To better understand the geometry and stress state and to assess the risk related to moderate-size earthquake faults, dense multi-parametric monitoring infrastructures referred to as Near Fault Observatories, have been deployed in the Central and Southern Apennines of Italy and across Europe over the last 15 years^7^.

In this study, we focus on the multi-segmented Irpinia fault system, in the Southern Apennines, that generated the 1980 Ms 6.9 earthquake^8^. This earthquake ruptured at least three fault segments, each of which were tens of km long over more than 40 s duration, resulting in a long-duration strong ground shaking that caused widespread building collapse and over 3000 fatalities^9^. The Irpinia region is classified as one of the highest seismic hazard areas in Italy (MPS Working group, 2004) with a relatively short return period for M 6 + earthquakes^10^, and a probability greater than 30% of a M5.5 + earthquake occurring within the next decade^11^.

**Fig. 1:**
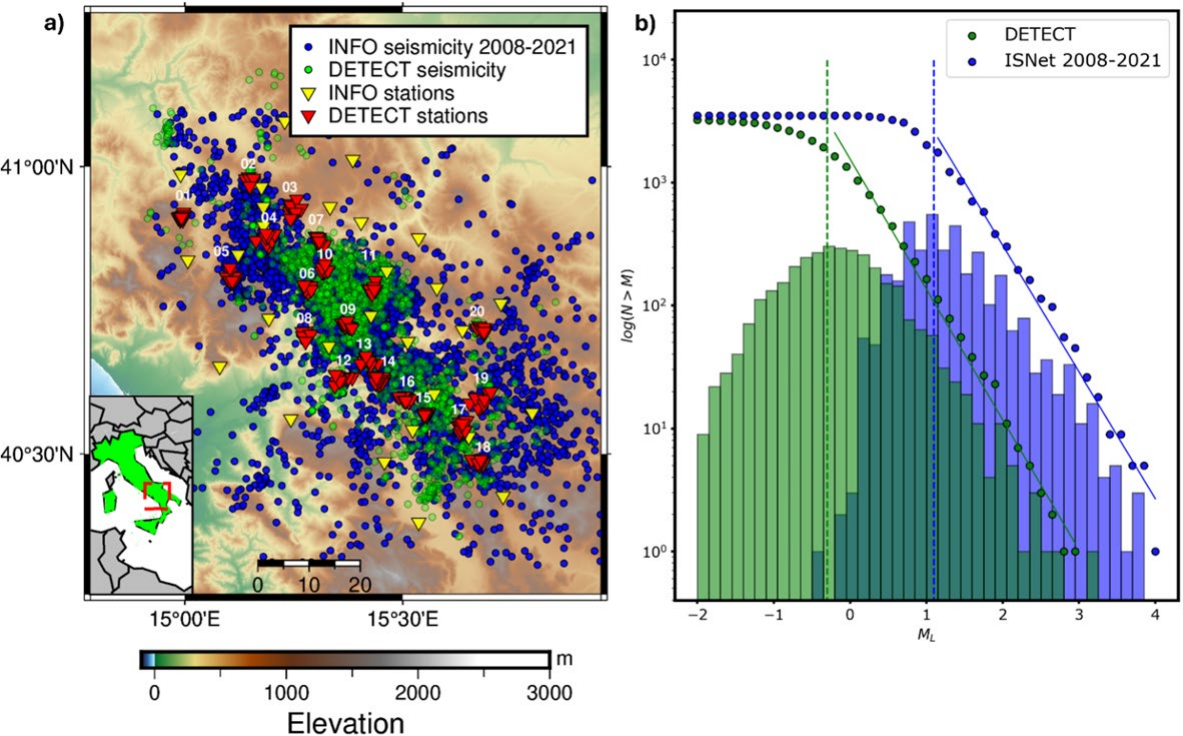
**Panel (a)**: Epicentral distribution of the earthquakes recorded by INFO (blue dots) within 2008 and September 2021 (start of the DETECT survey), along with the seismic stations (yellow triangles). Epicentral distribution of the relocated earthquakes occurred within the DETECT experiment (green dots), along with the stations of the dense arrays (red triangles). **Panel (b)**: Frequency-magnitude distribution for the earthquakes in the INFO catalog from 2008 to September 2021 (blue dots and bars) and analogous distribution for the earthquakes recorded within the DETECT survey (green dots and bars). The magnitude of completeness of the seismic catalog is improved by more than one unit (from $\:{\mathrm{M}}_{\mathrm{c}}$ 1.1 to -0.3), while we observe compatible b-values between the catalogs between decadal conventional and short-term dense monitoring. Maps have been generated using PyGMT (v0.9.0, https://www.pygmt.org/v0.9.0/).

In 2005, the Irpinia Near Fault Observatory (INFO) was created with the aim of developing a large research infrastructure in Earth Sciences to monitor the Irpinia fault system by a dense seismic network consisting of 39 stations, with inter-station distances ranging from 10 to 20 km^12^. This network allows for a local magnitude of completeness in seismic catalogs of $\:{M}_{l}\:$1.1^13^. The epicenters of the earthquakes occurring from 2008 through September 2021 are reported as blue circles in Fig. 1a, along with the stations of the INFO (yellow triangles in Fig. 1a). Background microseismicity appears to be sparse within the graben bounded by the main faults responsible for the 1980 Irpinia earthquake^14^ and occasionally clusters in sequences lasting for a few days, that rupture small, sub-parallel structures to those activated during the 1980 event^15,16^. Tomographic models^17,18^ suggest that the area is permeated by deep fluids, predominantly CO₂ and brine^19^. Additionally, geodetic data modeling reveals a non-linear elastic response of the shallow karst aquifers to hydrological loading, with the opening and closing of cracks correlated with fluctuations in seismicity rates in the area^20,21^. Long-term monitoring has provided evidence for structural segmentation and evolution of both crustal and source properties^22–25^.

Uncertainties in earthquake location, however, hinder the clear identification of causative fault structures, making it ambiguous whether the sparse hypocentral distributions are due to limitations in resolution or to a genuinely chaotic orientation and spatial distribution of small structures hosting the microseismic events^14,26^.

To investigate new technological solutions aimed at improving seismic monitoring capability, we deployed a constellation of 20 small-aperture seismic arrays, each consisting of 10 stations (200 stations in total) integrating INFO during the period September 2021 - July 2022, in the framework of a temporary experiment, named DETECT (DEnse mulTi-paramEtriC observations and 4D high resoluTion imaging, red triangles in Fig. 1a). DETECT is an international monitoring project coordinated by GFZ and the University of Napoli Federico II, that involved several Italian University and research institutes (INGV, CNR, Università di Salerno, and Università del Sannio).

Within the DETECT arrays, the average inter-station distance ranged from several hundred meters to one kilometer, while the average distance between arrays was approximately 10 km. Each array was equipped with one broadband seismometer, one 1 Hz sensor, and eight short-period (4.5 Hz natural frequency) geophones. The data collected were processed, standardized, and made publicly available.

The main goal of DETECT was the generation of enhanced catalogues of accurately located microseismic events. Machine learning (ML) and similarity-based methods have shown significant potential for increasing the size of seismic catalog by up to an order of magnitude, uncovering previously uncatalogued lower-magnitude events, even in areas for which the events were not included in the training dataset^27–29^. However, these approaches can suffer from a high rate of false positives and missed detections. Such limitations can be mitigated using well-designed ML models trained on region-specific datasets^30^ or by assisting more conservative ML detectors with robust, network-based similarity techniques (e.g^31^) , where events identified by ML algorithms can be used as templates for similarity-based detections^15^. Indeed, template matching algorithms target earthquakes occurring close to a known set of events and typically achieve a better detection performance for earthquakes reporting low signal-to-noise ratio^15,32,33^. Accurate hypocenter determinations of earthquakes in ML-enhanced catalogs have shown to provide significant insights into fault geometries^16,34–37^.

In this study, we characterized the enhanced seismic catalog, generated from the application of machine-learning (EQTransformer^28^, hereinafter EQT) and similarity-based (EQCorrscan^31^, hereinafter TM) detection strategies to the data collected by the DETECT survey. We then performed accurate hypocenter determination through advanced relative location techniques (HYPODD^38)^, comparing the seismic features revealed by the short-term catalog with the ordinary one derived over longer term. We investigated the relocated catalog analyzing the spatio-temporal characteristics of the seismic events, also in comparison with those from the long-term catalog, through clustering analysis and evaluated rupture propagation along the identified fault segments using numerical simulations, crucial to assess seismic hazard in the region.

## Results

### Spatial and statistical properties of the DETECT seismic catalog

The application of these techniques to continuous data from DETECT led to the identification of approximately 3,600 earthquakes that occurred during the 11-month experiment duration. This enhanced catalog (hereinafter the DETECT catalog) represents an ~ 8-fold increase in the number of earthquakes compared to the existing catalog provided by INFO within the same time window. The existing catalog is based on the use of a conventional network layout and seismic detection methods, as the visual inspection of records by operators analyzing data from the standard network stations. Focusing on the contribution of individual methodologies in detecting low-magnitude earthquakes, we found that the machine learning-generated catalog expanded the manual one by a factor of ~ 4. This improvement represents a twofold increase over the results reported by^15^ who applied the same deep learning detector to seismic sequences recorded by the INFO network. This highlights the efficacy of dense constellations of arrays in amplifying the detection capability of machine learning models for low-magnitude seismic events. Furthermore, the DETECT catalog, derived from advanced strategies applied to dense arrays, exhibits an earthquake count comparable to multiple years of conventional monitoring (Fig. 1b). The temporal distribution of the earthquakes (Figure S1) shows a reduced number of events between September and January, followed by an increase of the seismicity rate up to the end of the survey. The adopted detection strategy also allows for identification of near-overlapping events^15^, confirming the absence of significant seismic sequences during the experiment.

To characterize the earthquakes in the DETECT catalog, we performed accurate relative event relocation. Refinement of phase arrival times using hierarchical clustering^39^ yielded average corrections within ~ 0.1 s for both P and S picks (Figure S2). This result not only supports the high accuracy in the automatic identifications of phase arrival times but also provides critical adjustments for constraining the fine-scale properties of seismogenic sources. As a main methodological outcome, we successfully relocated 2,248 earthquakes (~ 65% of the detected events), nearly doubling the fraction of relocated events in enhanced catalogs for seismic sequences^16^ and significantly surpassing typical relocation percentages in template-matching-derived catalogs^36,40^. This improvement is attributed to the deployment of the dense seismic arrays, which facilitates the detection of a larger number of phase arrival times, even for low-magnitude events (~ 47,000 P-wave and ~ 72,000 S-wave picks). The increased number of picks resulted in a substantial dataset of differential travel times, which are fundamental to achieve high-precision earthquake locations. Location uncertainties evaluated with a singular value decomposition inversion strategy^38^, which provides reliable error estimates by accounting for covariances, are characterized by median horizontal and vertical uncertainties of 130 m and 120 m, respectively, with 80% of the relative locations constrained within 250 m (See Methods, Figures S3, S4).

The left panel of Fig. 1 illustrates the epicenters of the relocated earthquakes, showcasing the spatial distribution of the relocated seismicity observed during DETECT (shaded green points) compared to the seismicity detected by INFO (blue points) from 2008 up to the start of the dense array monitoring (September 2021).

The spatial distribution of epicenters from the DETECT catalog reveals variable seismicity levels across different sectors of the Irpinia region. Most of the seismicity is concentrated in the Central and Southern sectors, while the Northern sector exhibits significantly lower activity, predominantly characterized by isolated events and a single swarm-like sequence of 30 earthquakes associated with a $\:{M}_{l}\:$2.1 event located outside the coverage area of DETECT (northernmost green cluster in Fig. 1a). When comparing the seismicity observed during the experiment with that recorded by INFO between 2008 and September 2021 (the start time of the experiment), we find consistency in the distribution of hypocenters. This highlights the persistent occurrence of microseismicity in areas prone to generating moderate-to-low magnitude earthquakes along the Irpinia fault system. Most of the earthquakes recorded by the dense arrays occur in regions reporting a high density of events already identified in the long-term manual catalog, as confirmed by the comparison of the density maps for the declustered catalogs^41^ evaluated at various depths (Figure S5). These maps feature zero-lag cross-correlation coefficients above 0.7, with a similarity value of 0.9 between 5 and 9 km of depth. We used the Chamfer distance^42^ to measure the connection between relocated hypocenters from this analysis and the seismicity depicted by the long-term INFO catalog. From the spatial distribution of the Chamfer distance, we observed lower values of the metric in the Central sector of the area, below 1 km. The distance increases in the Northern sector, which is scarcely populated by earthquakes in the DETECT catalog, while the metrics assume intermediate values when moving towards the Southern sector (Figure S6). We retrieved a median value of 1.2 km for the entire area, which is significantly lower than the median Chamfer distance (1.7 km) from the comparison between the short-term INFO catalog (INFO catalog during the DETECT deployment) and the long-term INFO catalog. The lower distance resulting from the DETECT catalog supports the integration of array deployments to better resolve the structures illuminated by conventional monitoring infrastructures. Moreover, an absence of seismicity is evident in the Southeastern edge of the region (Fig. 1a), a feature previously reported by^14^ inspecting a catalog of six years of microseismicity with moment magnitude ranging between 0.9 and 3.1. This phenomenon has been attributed to the presence of a contact-zone between geological units with differing rheological properties in response to the NE–SW stress regime acting on the chain. These results demonstrate that intensive short-term monitoring can discern features that would otherwise only emerge after many years of standard monitoring.

To evaluate the statistical parameters of the DETECT catalog, we calculated the local magnitudes of the events using a scale calibrated for the area^43^, resulting in values ranging in $\:{M}_{l}$ [-1.9 to 2.9]. We estimated the statistical parameters of the Gutenberg-Richter distribution for the earthquakes in the DETECT seismic catalog, including its magnitude of completeness (Mc) following the method of^44^. Figure 1b illustrates the Gutenberg-Richter distribution for the DETECT catalog (11 months) compared to the INFO catalog spanning 13 years of conventional monitoring. Both distributions are plotted with overlaid discrete magnitude bins for direct comparison. The analysis of the completeness magnitude reveals that monitoring seismogenic sources in Irpinia using temporary dense array deployments significantly reduces the detection and completeness thresholds. Specifically, Mc​ decreases by 1.4 magnitude units, down to -0.3 for the DETECT catalog, as compared to corresponding value characterizing the catalog from conventional monitoring^13^. Moreover, the comparison of the b-values for the two catalogs shows consistent slopes of the Gutenberg-Richter distribution, suggesting scale-invariant seismic generation processes from $\:{M}_{l}$ 0 to 4. Specifically, we found b_DETECT_ = − 1.06 ± 0.10 ​and b_INFO_ = − 1.03 ± 0.08, indicating that the temporary deployment of dense arrays not only enhances detection capabilities but also provides robust statistical consistency with long-term monitoring data, downscaling statistical features of the seismicity to a shorter temporal scale and smaller magnitude events.

### Depth-dependent seismicity pattern

To identify spatial and temporal patterns of seismicity, we conducted a DBSCAN clustering analysis^45,46^ imposing a minimum of 10 events to declare a cluster of earthquakes. This analysis identified 22 seismicity clusters, with the three largest clusters comprising approximately 100 earthquakes each. These events occurred in the Central and Southern sectors as part of three swarm-like sequences culminating in events smaller than $\:{M}_{l}\:$2.0.

**Fig. 2:**
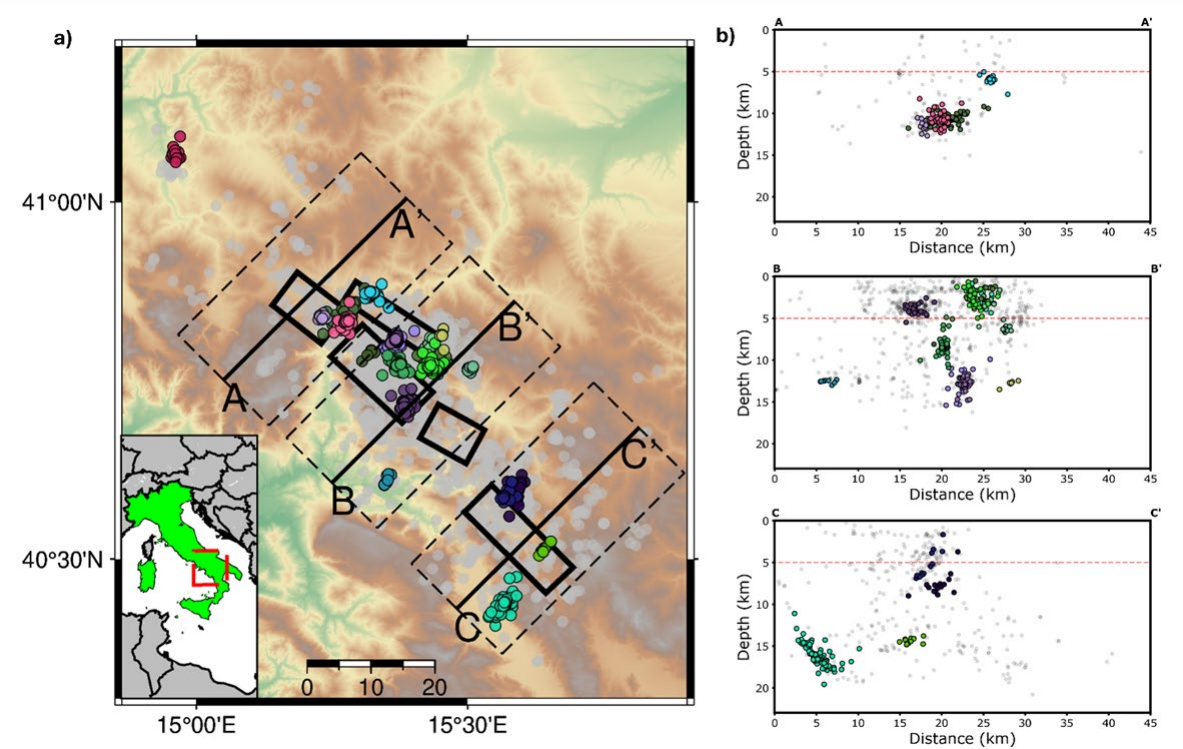
Clustering analysis of DETECT seismicity using DBSCAN. **Panel (a)**: Epicentral distribution of clustered (colored dots, 22 clusters with a minimum number of 10 earthquakes) and isolated (shaded gray dots) seismicity. Black boxes indicate the main seismogenic structures in the area. **Panel (b)**: Cross-section representations of relocated hypocenters for the seismicity in the Northern (Section A-A’), Central (Section B-B’) and Southern (Section C-C’) sectors, color coded according to clustered and isolated seismicity. Each cross-section includes earthquakes within ± 10. Most of the earthquakes occurring at depth > 5 km belong to a seismic cluster (45%), while the percentage drops to 20% for shallower earthquakes. Maps have been generated using PyGMT (v0.9.0, https://www.pygmt.org/v0.9.0/).

Analysis of the characteristic depths of clustered seismicity revealed a distinct contrast between deep and shallow seismicity. We set the separation depth between the two classes to 5 km, at the top of the Apulian platform as depicted by tomographic models^14^. Shallow seismicity appears sparser and lacks systematic clustering in space and time, while deeper seismicity predominantly forms spatially compact concentrations. Approximately 45% of earthquakes deeper than 5 km are part of a cluster, compared to only 20% for shallower events. Figure 2 illustrates the clustered seismicity alongside characteristic cross-sections showing the relocated hypocenters. The events are projected on vertical planes oriented according to the orthogonal direction to the strike of the fault segments generating the 1980 Irpinia earthquake^8^. Figure 2 shows seismicity clusters in the Northern, Central and Southern sectors, (section A-A’, B-B’ and C-C’, respectively), including events within $\:\pm\:$10 km from the vertical planes, with isolated earthquakes represented as shaded black dots. Most clusters are concentrated in the Central Irpinia sector, where the most abundant families occur at depths between 8 and 15 km, consistent with typical earthquake depths in the Southern Apennines^16,22,47^. In contrast, the Southern sector hosts deeper clusters, with some earthquakes occurring below 15 km. Notably, the larger cluster, consisting of 110 events, is located in this sector (cyan dots in Fig. 2b-Section C-C’) and illuminates a $\:\sim\:5$ km-long deep structure, west of the main Irpinia fault zone. The results were proved robust against variations in the separation depth between shallow and deep seismicity and the key parameters in the cluster definition (see Methods and Figure S7).

## Discussion and conclusions

A main methodological outcome of the DETECT experiment is that deploying a constellation of dense seismic arrays combined with advanced detection techniques significantly enhances the seismic catalog as compared to the sole use of conventional networks, while improving the quality and accuracy of earthquake locations and source parameter determinations. By integrating advanced detection techniques with a high-density station deployment over one year, we achieved large catalogs comparable in size to those produced by more than a decade of continuous monitoring using conventional local seismic networks, while maintaining a location accuracy comparable to that obtained from the relocation of larger magnitude events in the long-term catalog (~ 100 m;^26)^. To isolate the enhancement carried by seismic arrays, we considered the findings by^15^ on seismic sequences in the area recorded by the permanent Near Fault Observatory. Our results indicate that the use of arrays can increase the number of events detected by machine learning techniques by a factor of 2 to 4 as compared to the ordinary network. This enhancement in the catalog suggests the integration of arrays in near-fault observations to have access to smaller rupture scales and its temporary deployment in areas not monitored by permanent dense infrastructures.

The DETECT experiment reveals a stable pattern of seismicity throughout the considered period, consistent with long-term monitoring, in terms of statistical properties, characterized by the *b*-value of the Gutenberg-Richter relationship, with the magnitude of detected events systematically decreasing by 1 to 1.5 units. Density plots support similarity of the spatial distribution of earthquakes, highlighting the spatial invariance of seismic activity on a shorter time scale, especially in the Central and Southern sectors. Therefore, statistical characteristics of seismicity (e.g., productivity and ratio between small and large events) can be downscaled from rupture sizes of approximately 100 m, typical of standard near-fault catalogs, to the 10-m scale of the earthquakes identified in this study.

We identified a separation between shallow and deep earthquake behavior, with a discriminating depth around 5 km, suggesting distinct physical mechanisms governing their occurrence. Shallow events appear sparsely located and are primarily concentrated in the central part of the region within the volume enclosed by two boundary faults of the 1980 Irpinia earthquake^14^. Here, hydrological loading was shown to influence the stress field in karst aquifers, inducing seasonal oscillations of strain along the NE-SW direction, perpendicular to the trend of the Apennines^20,21^. In this context, seismicity may be linked to the opening and closing of fluid-permeated microcracks, generating localized earthquakes with a sparse spatial distribution. Additionally, the shallow portion of this area exhibits a lower-than-average stress drop^24^, highlighting the strong influence of fluids—likely brine and CO₂ on stress release mechanisms^19,22^.

Deeper events show a more clustered pattern, with small sequences characterized by low magnitude mainshocks followed by aftershocks. Most of the identified clusters extend over a few hundred meters and appear more confined as compared to the size of moderate seismic sequences in the area, where seismicity evolved within larger kilometric-scale patches^17^. Aftershock magnitude generally falls within the 0–1 magnitude range, corresponding to rupture extent of approximately 5–15 m, according to the self-similar, constant stress drop scaling from earlier studies on the area^16,24,47^. This observation supports the idea that seismicity in the area is primarily driven by stress release following strain accumulation during the inter-seismic period, within a high-fractured medium, with localized fractures nearby or along the major structures. Many of these events appear isolated when recorded by the standard network, which suffers from lack of resolution, required to identify such deep and small aftershocks whose signals remain obscured by background noise. This prevents the reconstruction of the sequences in their full complexity, which is crucial for understanding their generation mechanisms and, on a larger scale, the characteristics of the seismic cycle of the fault zone across multiple scales.

**Fig. 3:**
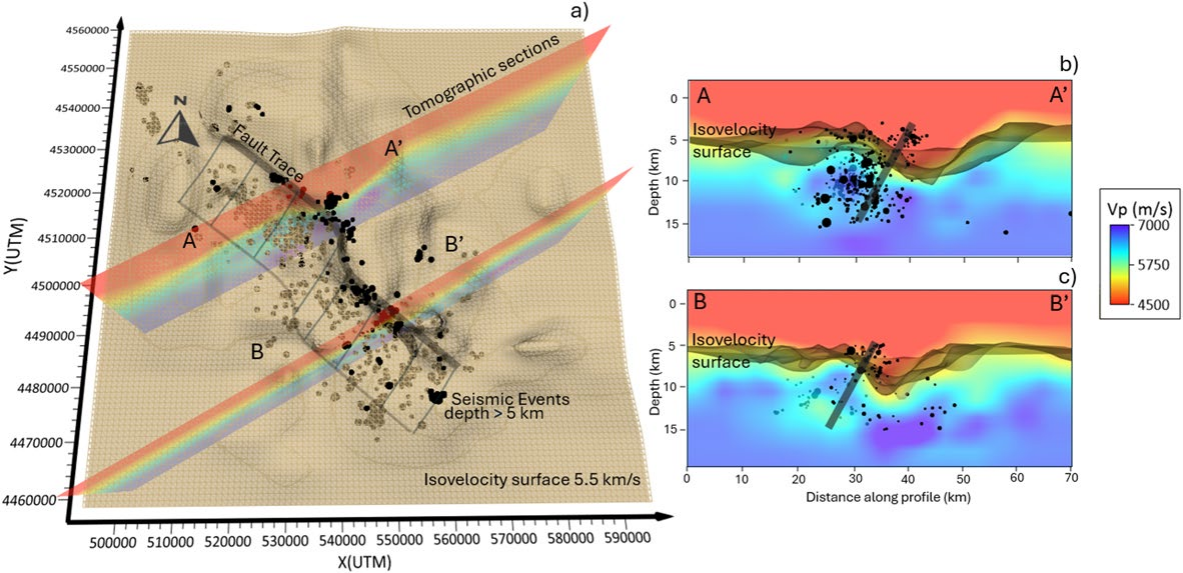
A comprehensive view of the fault trace reconstructed by integrating the enhanced micro-seismicity DETECT catalog (panel **a**, events with depth greater than 5 km are represented by black dots if above the iso-surface and brown dots if below) and the P-wave velocity tomography^22^. Here we show the iso-velocity surface at 5.5 km/s and two vertical sections (panel **b**) crossing the fault surface at the boundary of the low velocity anomaly (red shaded area). The thickness of the fault surface is compatible with estimated location uncertainties. This figure was constructed with Surfer from Golden Software, LLC (version 27, www.goldensoftware.com).

When interpreting seismic events jointly with the 3D tomographic model of^22^, part of the seismicity occurs along the structure described as a step-over in^26^, with a right-stepping bend. (Fig. 3). To the southeast, seismicity extends well into the Apulian carbonate platform, aligning with and surrounding a previously identified, southeast-dipping, long-lived and reactivated major fault (Fig. 3, S8^17,48)^. Northward shallowing of high v_p_ velocity zones in the tomographic model delineates such a bend, where several events in the DETECT catalog are located. This bend can be interpreted as the basement culmination in the hanging wall of this segmented, reactivated fault. This interpretation is further supported by Bouguer gravity anomaly data for the study area (e.g^49)^. Whether this whole structure directly accommodates the observed seismicity or whether the events occur on nearby unconnected, sub-parallel small-scale faults within a highly fractured medium—while the main fault remains locked—requires further investigation. Given its total length of 50–60 km, this fault has the potential to generate an event of up to *M* 7.0^8,50^ in cases when rupture propagates across the fault offset and involves the entire fault system.

To assess whether a rupture can dynamically propagate across the bend, we conducted 2D numerical simulations using a spectral element solution of the antiplane elastodynamic equation ^51,52^. We assumed a regional stress field with the maximum principal stress oriented vertically and the minimum compressive stress directed horizontally, perpendicular to the Apennine chain^53^. When projecting this stress field onto the segmented fault system, the two longest segments appear well-oriented for rupture propagation^53^, whereas the oblique segment is less favorably aligned for rupture continuation.

**Fig. 4:**
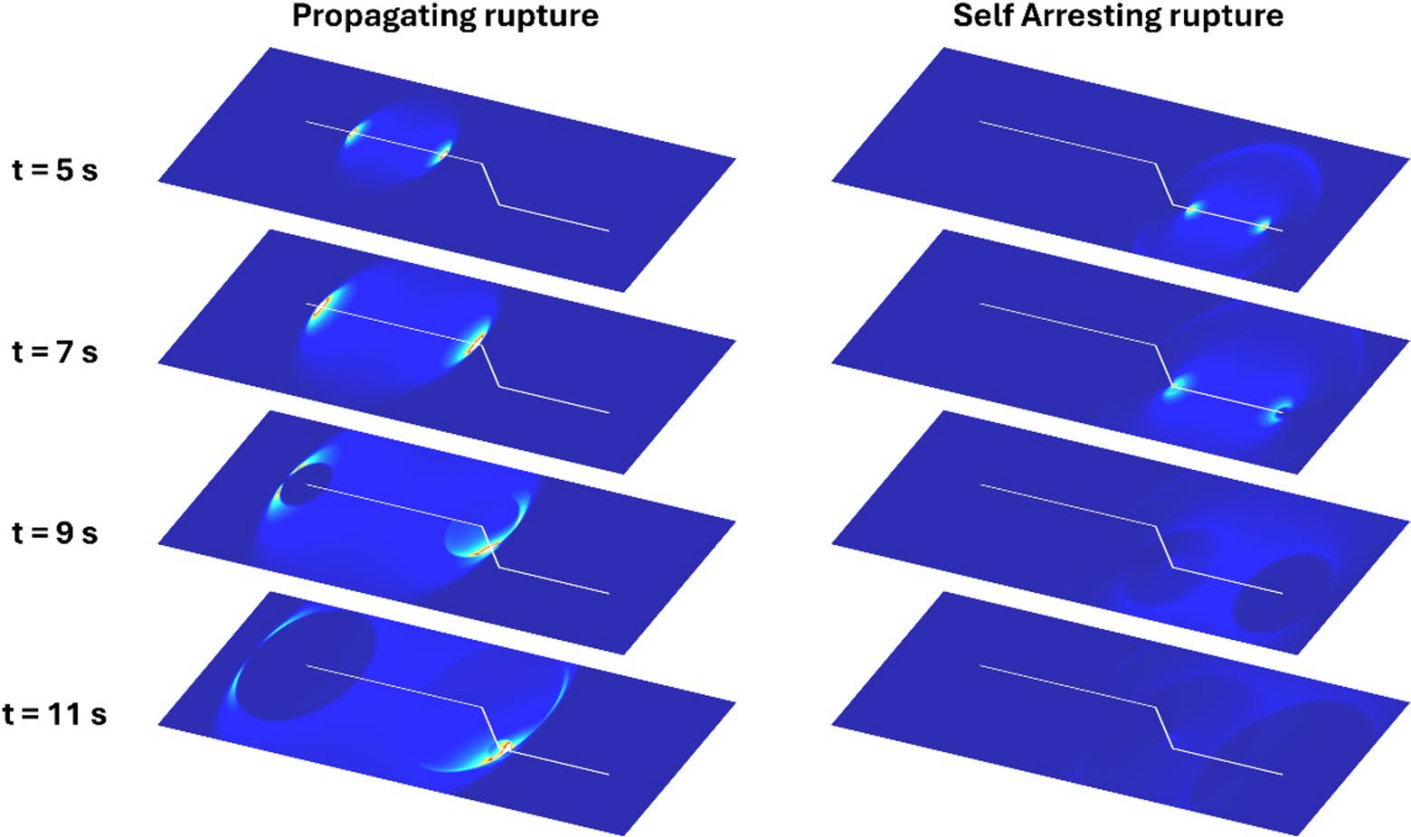
Examples of dynamic antiplane ruptures that propagate across the bend (left panel) or arrest after turning the kink (right panel).

We explored rupture scenarios by varying key parameters, such as the main stress components, frictional coefficients, and rupture nucleation locations. Illustrative examples of rupture evolutions under these conditions are presented in Fig. 4. Our findings reveal that, in most tested cases, the rupture propagates through the entire segmented fault structure, highlighting a significant potential for generating large M6.5 + earthquakes. However, under conditions where the stress drop along bended fault segment remains limited (a few percent of the available strength excess), rupture tends to arrest at the kink. This occurs because the available elastodynamic energy becomes insufficient to overcome the fracture energy needed for frictional weakening and continued rupture propagation^54^. We also tested scenarios considering a step-over without mechanical fault connection. In this case the rupture is impeded to jump from one segment to the other, except for secondary segments close to critical state (Figure S9).

Further investigations are required to refine these rupture scenarios and better quantify seismic hazard. These should include detailed characterization of stress fields and frictional conditions through laboratory and field measurements, coupled with advanced 3D numerical modeling to systematically evaluate rupture sensitivity to fault geometry and stress field complexities. In this respect, high-resolution seismic catalogs could be leveraged to constrain frictional heterogeneity and stress-state variations along fault segments. Combining these observational and modeling approaches will help reduce uncertainties in hazard assessments and contribute to reliable earthquake rupture forecasts.

## Methods

### Earthquake detection

We followed the same earthquake detection strategy of^15^ for seismic sequences in the Irpinia region. The workflow is grounded on the use of the machine learning detector EQTransformer^28^, which provided a diverse set of templates to be used as the basis for further similarity-based detection using the template matching technique EQCorrscan^31^. After splitting the network into 6 subnetworks of 6 arrays each, with an overlapping of 3 arrays between consecutive subnetworks, we performed earthquake detection independently for each subnetwork and integrated the declaration among the subnetworks according to the detection times. For each subnetwork, we applied EQTransformer on daily continuous data streams, resampled to 100 Hz and filtered in the frequency band^1–45^ Hz using the parameterization of^15^, requiring a probability value of 0.1 for P and S arrival times and using 50% overlap between consecutive time windows. Detections were declared when at least 5 picks were associated within time windows of 10 s and were visually confirmed. The machine learning catalog was used as template set for a similarity search using EQCorrscan. Each template event contained only the picked stations, selecting 1.6 s long windows extracted around the automatic picks (including 0.15 s of pre-pick waveforms). We decimated the traces to 25 Hz and filtered in the frequency band^2–9^ Hz. For the similarity detection threshold, we selected the sum of cross-correlation coefficient (SCC) between the portion of continuous streams and the templates. We declared an event when the SCC overcomes a similarity threshold with at least one template, fixed at 8 times the MAD of the cross-correlation coefficients between the template and the one-hour chunk of continuous streams. Moreover, we performed cross-correlation picking for the detections, requiring a minimum similarity coefficient of 0.6 at channel level. We applied the automatic selection criteria of^15^ for limiting the false declarations inside the catalog and visually inspected the remaining declarations.

### Event location and magnitude

Using automatic picks, we performed a preliminary location of the earthquakes. We used NLLoc^55^ and a 1D layered velocity model tailored for the area^56^ for retrieving the absolute location of the events. For these initial locations, we determined location uncertainties of few kilometers for horizontal and vertical locations and decimals of seconds for the time residuals.

To improve the quality of the automatic picks and obtain high quality double difference locations, we applied a refined picking procedure based on cross-correlation and hierarchical clustering^39^. The refinement picking procedure was performed at each individual station by correlating waveforms around the picks of events located in 20 km x 20 km squares. Seismic records were bandpass filtered^1–15^ Hz and polarized using a polarization filter^39,57^. We correlated the seismic waveforms on the three components independently in a time window of 0.25 s around the initial picks. The normalized cross-correlation coefficient threshold to define the family was set to 0.8. 10,657 P-wave picks and 34,924 S-wave picks survived the refined picking procedure.

We located the hypocenters with NLLoc^55^ in 3D velocity models optimized for the area^22^. The refined picks allowed an increase in the accuracy of the absolute location by reducing the median location error from 1 km to 0.4 km (Figure S10) and the median root-mean-square (RMS) from 0.2 s to 0.1 s (Figure S10). We computed the cross-correlation (CC) differential times based on the refined locations. Finally, we relocated the seismicity with HypoDD^38^ including both the CC and catalog differential times. The CC differential travel times were calculated between events within families built during the refinement picking procedure.

To estimate final location uncertainties, we used the singular value decomposition (SVD) factorization implemented in HypoDD, which provides reliable least-squares error estimates through covariances^38,39,58^. Since this approach is limited to small and well-conditioned event clusters, we constructed a representative subset of 290 events to be analyzed with SVD by maximizing the number of events while ensuring that the distribution of azimuthal coverage (GAP, see Figure S3-right panel) and the number of differential times (see Figure S3-left panel) mimic those of the entire catalogue.

We computed the local magnitude for equivalent Wood-Anderson displacement, obtained from integration of velocity waveforms, assigning absolute locations to those that were not relocated, using the empirical local magnitude relationship of^43^.

### Event clustering

We used DBSCAN^45^ to identify seismicity clusters within the relocated DETECT catalog. For clustering seismicity, we also included the event occurrence time in the metrics. We selected the maximum normalized Euclidean distance for considering two points as neighbors in space and time $\:ϵ$=3 (spatial distance was normalized to 1 km, time distance to 1 day) and the number of samples in a neighborhood of a point to be considered as a core point min_samples = 10. This parameterization was calibrated to successfully identify clustered seismicity occurred within three seismic swarms during February 2022. We also explored variations in $\:ϵ$ between 1.5 and 4.5 and min_samples between 7 and 15, to support the robustness of the different clustering fractions of shallow and deep seismicity (Figure S7).

### Numerical simulations

We conducted numerical simulations using the spectral element code developed by^51^, modified to model 2D antiplane ruptures. We discretized the elastic medium using quadrangular elements, with an eighth-order polynomial approximation to solve the elastodynamic equations within each element. We included perfectly matched layers in the modelling to prevent wave reflections at domain boundaries^59^. The fault consists of three branches, measuring 32 km, 9.6 km, and 20 km in length, respectively. We imposed a remote regional stress field with the maximum principal stress s_1_ oriented vertically and the minimum compressive stress s_3_ aligned horizontally, perpendicular to the trend of the Apennines^53^. The two parallel fault branches have a strike aligned with the intermediate stress direction s_2_, while the oblique segment forms a 35°S angle relative to s_2_​ direction (Fig. 4). We projected the stress field onto the three segments assuming a fault dip of 60°.

We explored s_1_ values between 60 and 80 MPa, s_3_​ values between 20 and 30 MPa, a static friction coefficient ranging from 0.6 to 0.7, and a dynamic friction coefficient between 0.25 and 0.35. These parameters were constrained by the maximum lithostatic load in the region and the expected stress drops, which range between 1 and 15 MPa, as inferred from previous events^24,60^. The intermediate stress s_2_​ was set based on the R value from^53^. Rupture nucleated by locally increasing the initial tangential stress above the failure threshold within a small patch, whose size was comparable to the nucleation length defined by^61^. We modeled the fracture process using a linear slip-weakening law, where stress decreases from the yield level to the dynamic level over a critical slip distance D_c_=1 m.

## Supplementary Information

Below is the link to the electronic supplementary material.

Supplementary Material 1

## Data Availability

Seismic waveforms recorded during the DETECT experiment are available and can be accessed at http://eida.gfz.de/webdc3/ under the network code ZK (The Irpinia Seismic Array). Metadata information can be retrieved at https://geofon.gfz.de/doi/network/ZK/2021^62^ (accessed on 2025-12-05). Earthquake detection is performed using EQTransformer (https://github.com/smousavi05/EQTransformer, accessed on 2025-05-09) and EQCorrscan (https://github.com/eqcorrscan/EQcorrscan, accessed on 2025-05-09). Earthquake relocations were performed using NonLinLoc (http://alomax.free.fr/nlloc/soft5.00/index.html, accessed on 2025-12-03) and HYPODD (https://github.com/fwaldhauser/HypoDD, accessed on 2025-05-09). The DETECT seismic catalog is available at the following link: https://zenodo.org/records/15372023. INFO catalog is available at the Irpinia Near Fault Observatory website (http://isnet-bulletin.fisica.unina.it/cgi-bin/isnet-events/isnet.cgi) and at the EPOS Data Portal (https://www.ics-c.epos-eu.org/) - IRPINIA Seismic Events provided by Università di Napoli Federico II (accessed on 2025-12-03). Maps and images in Figs. 1, and 2 were produced using PyGMT^63^ v0.9.0 and Matplotlib^64^ v3.10.7. Figure 3 was constructed with Surfer from Golden Software, LLC (version 27, www.goldensoftware.com), used under an educational license.
