# Supplementary material for: Enhancing the resolution of microseismicity through dense array monitoring in complex extensional settings
Source: Sci Rep. 2026 Jan 17;16:5639. doi: 10.1038/s41598-026-35586-3 (PMC12891549; doi:10.1038/s41598-026-35586-3)
Supplement: Supplementary file 1 — Supplementary Material 1 [file 41598_2026_35586_MOESM1_ESM.docx]

*Supplementary Information for:*

**Enhancing the resolution of microseismicity through dense array monitoring in complex extensional settings**

**Francesco Scotto di Uccio, Titouan Muzellec, Antonio Scala, Grazia De Landro, Giovanni Camanni, Francesco Carotenuto, Luca Elia, Matteo Picozzi, Aldo Zollo, Claudio Strumia, Gregory C. Beroza, Gaetano Festa**

**Figure S1**


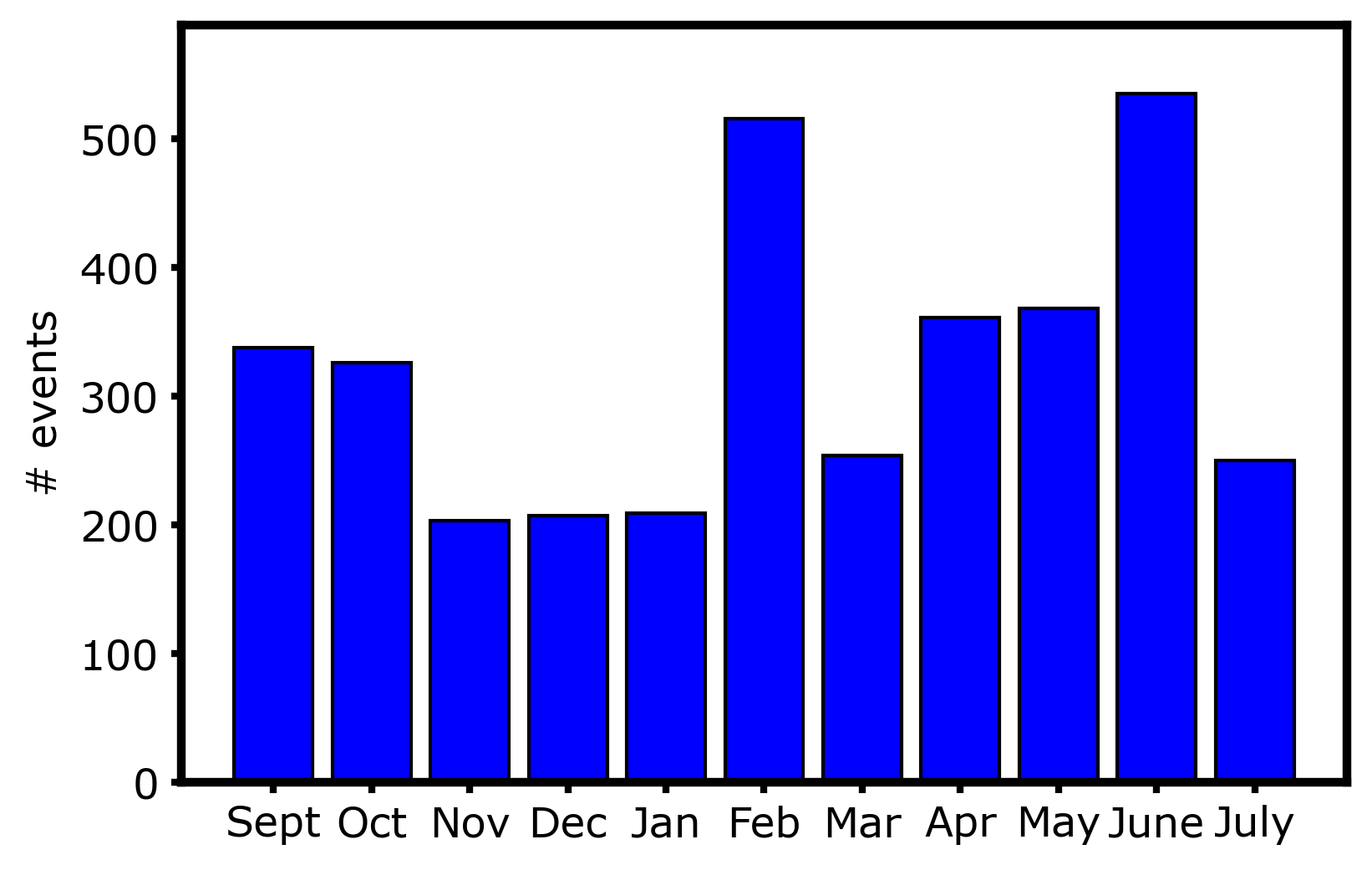


***Figure S1:*** *Monthly distribution of the earthquakes in the enhanced DETECT catalog detected by the integration of machine learning and similarity-based techniques. Low seismicity rate is observed from September to February, while we identified more earthquakes towards the end of the survey.*

**Figure S2**


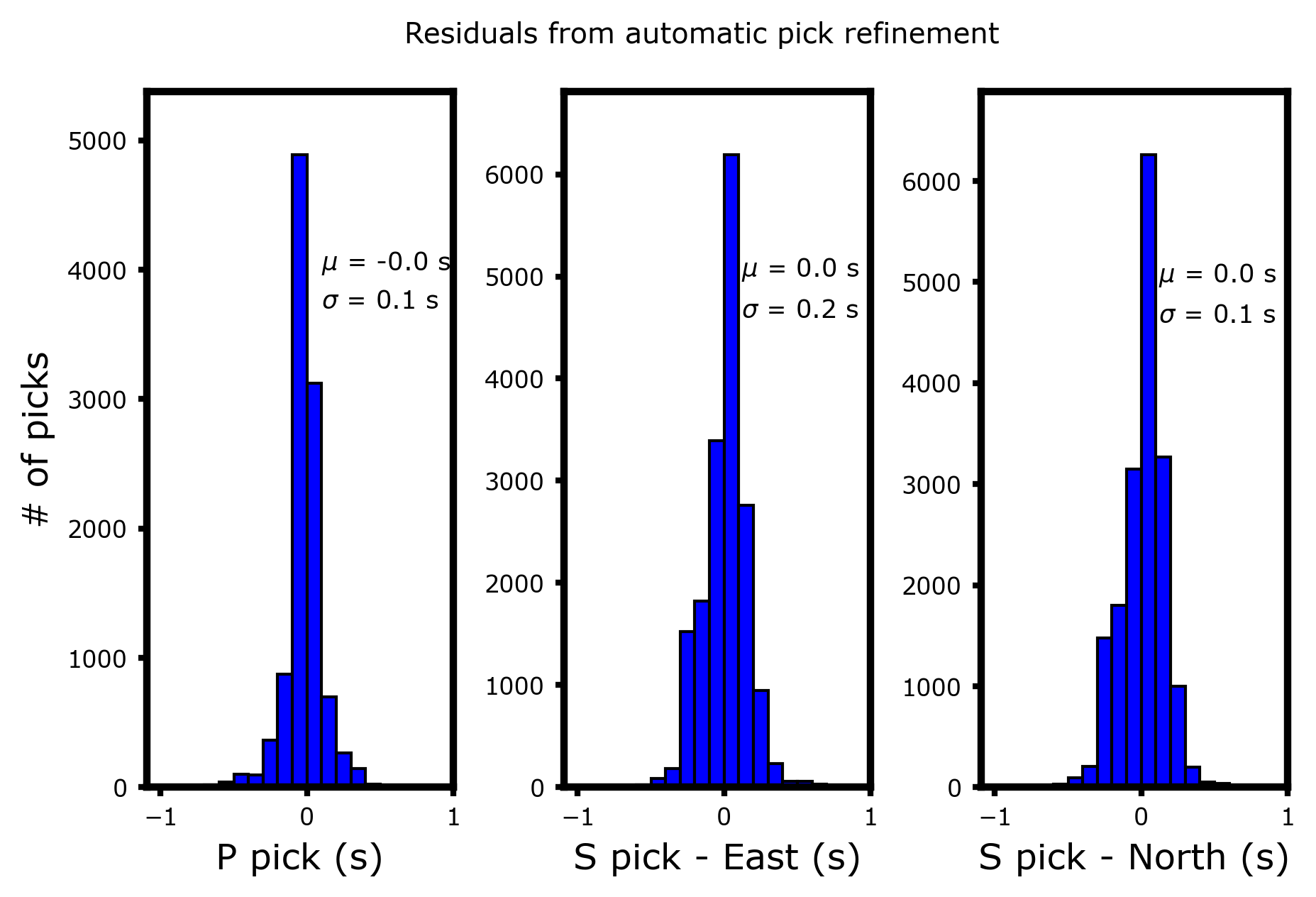


***Figure S2:*** *Results from the refinement of the automatic phase arrival times using waveform similarity and hierarchical clustering for the P and S picks. The average correction term is compatible with 0 for both phases, however small corrections can be introduced for constraining small-scale features of seismicity.*

**Figure S3**


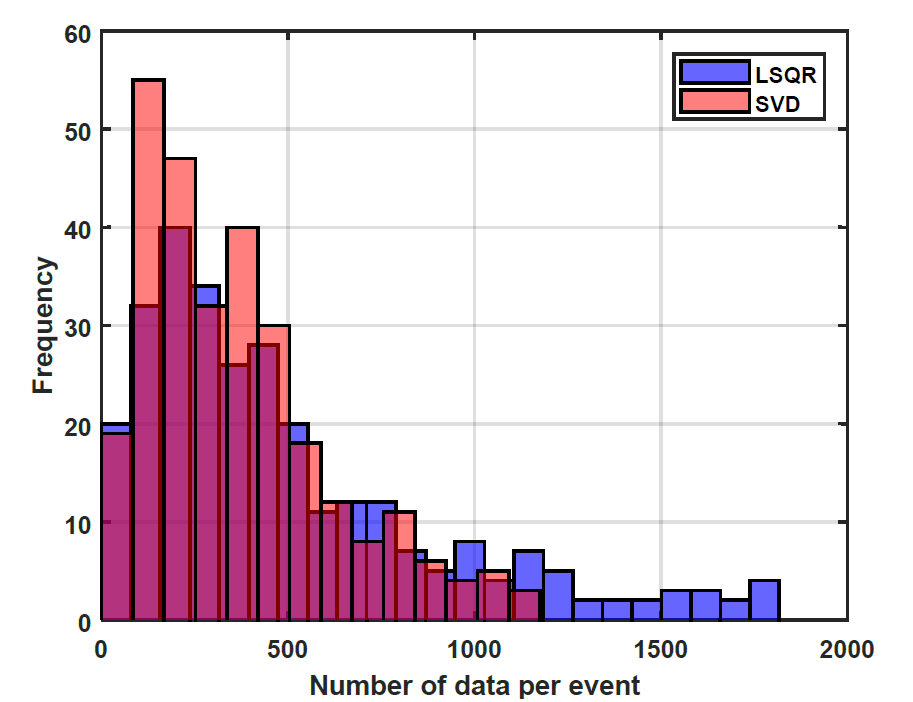

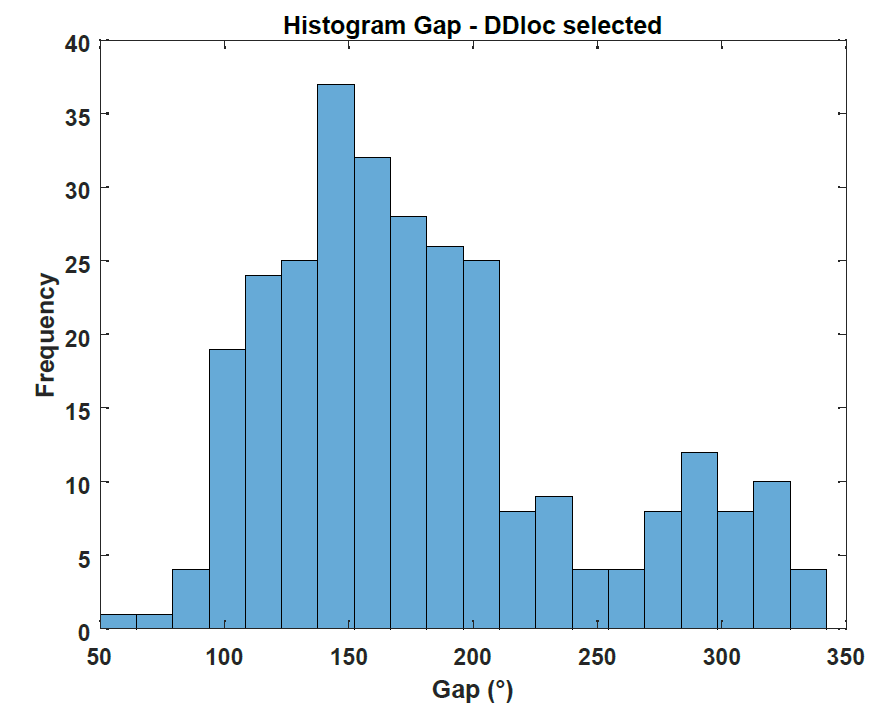


***Figure S3:*** *Left panel) Histogram of the number of differential data used in the location of the entire catalogue and the SVD subset. Right panel) GAP distribution for the subset of located events with SVD.*

**Figure S4**


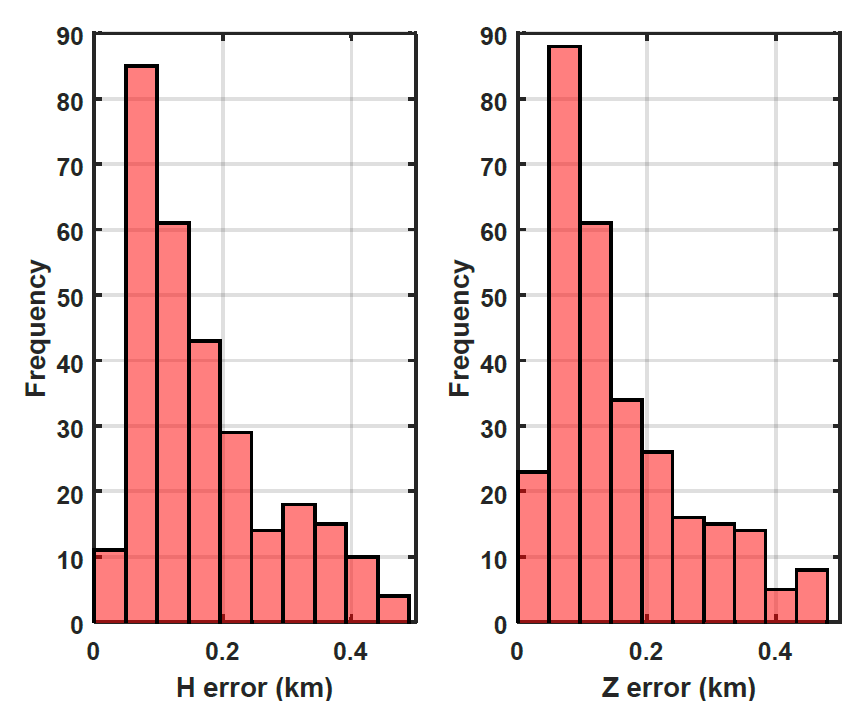


**Figure S4*:*** *Histogram of location uncertainties for the selected events in the SVD inversion. The results indicate that 80% of these events show horizontal and vertical uncertainties below 250 m, providing a representative estimate of the error distribution for the entire catalogue. Median horizontal and vertical uncertainties are 130 m and 120 m, respectively.*

**Figure S5**


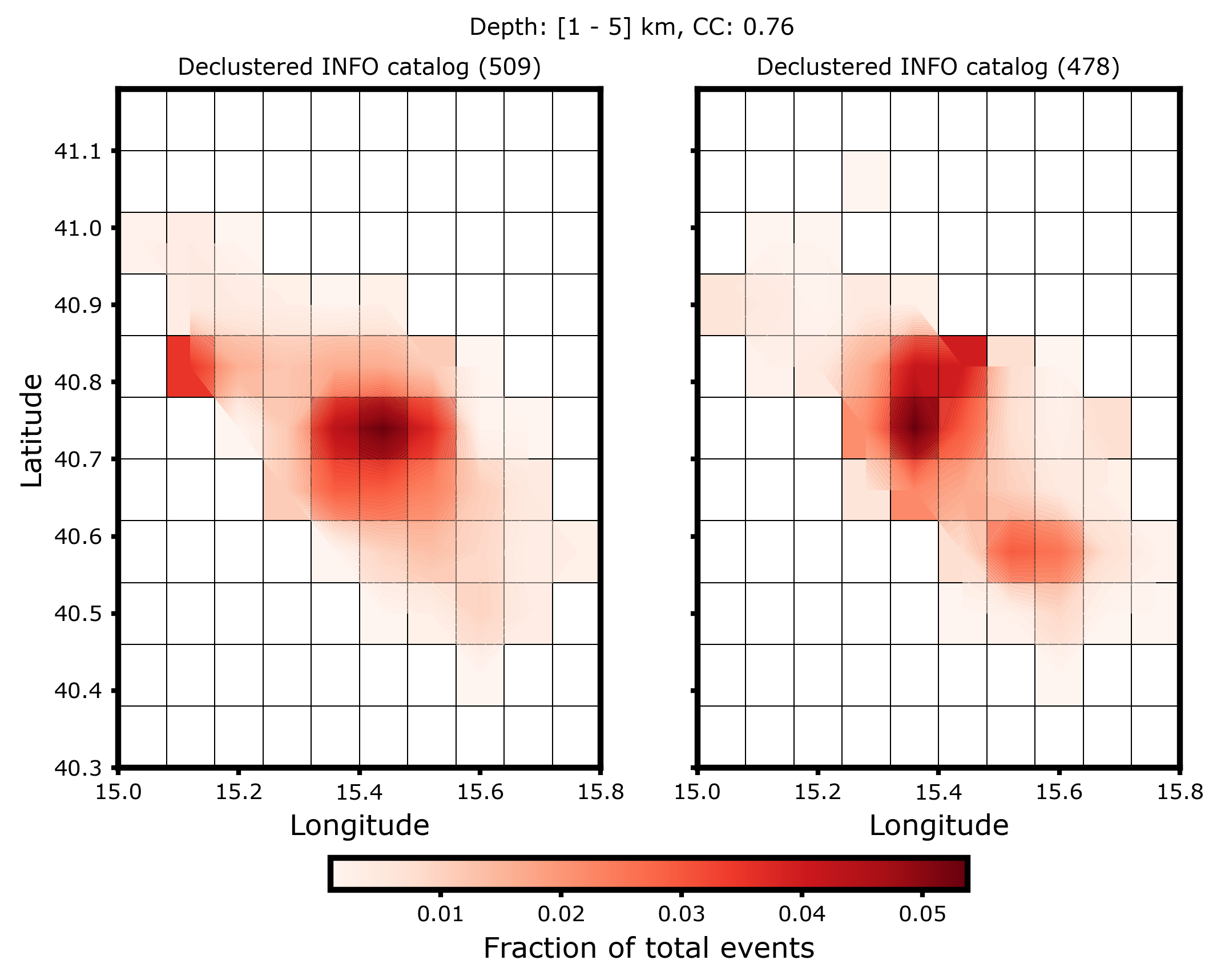

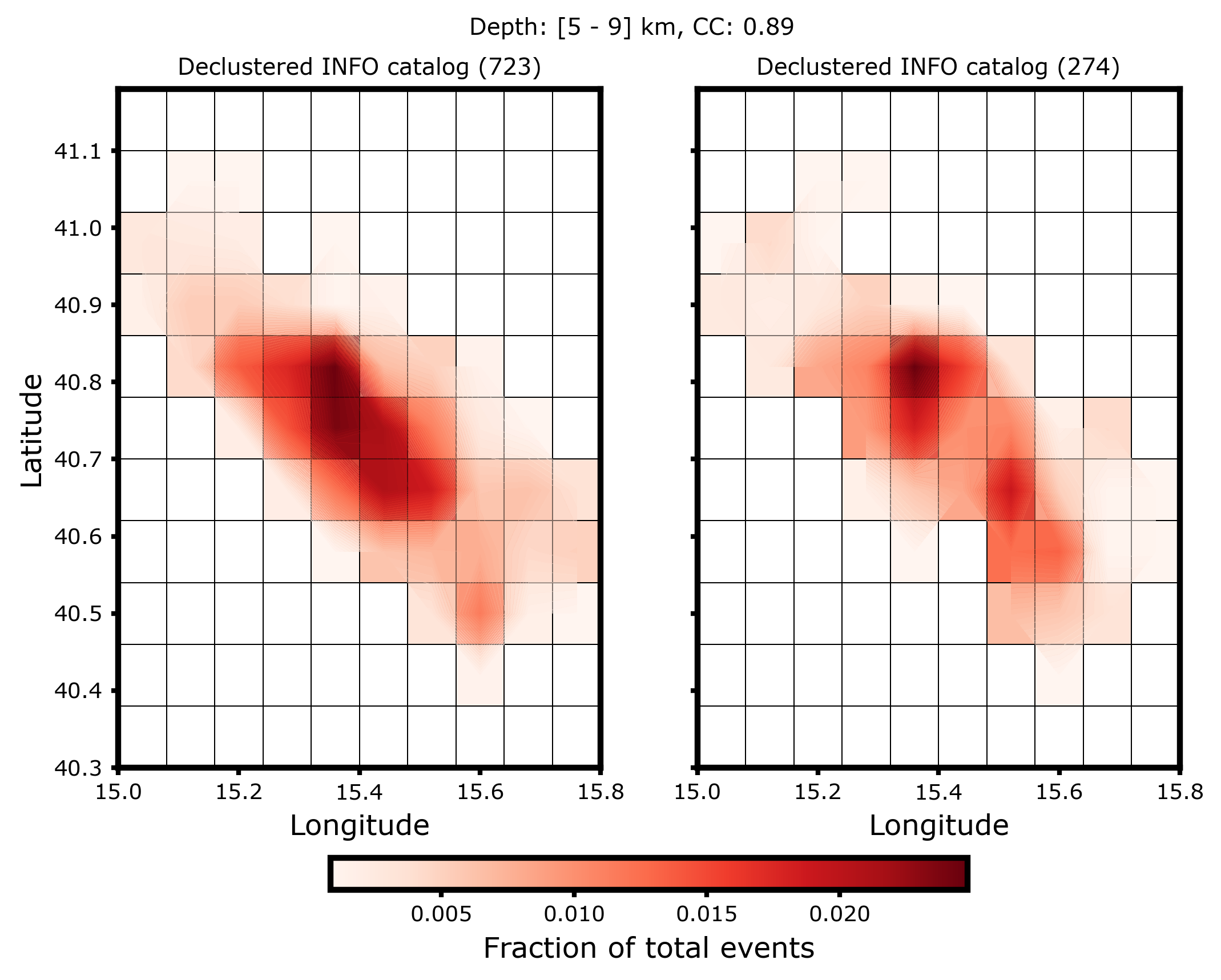


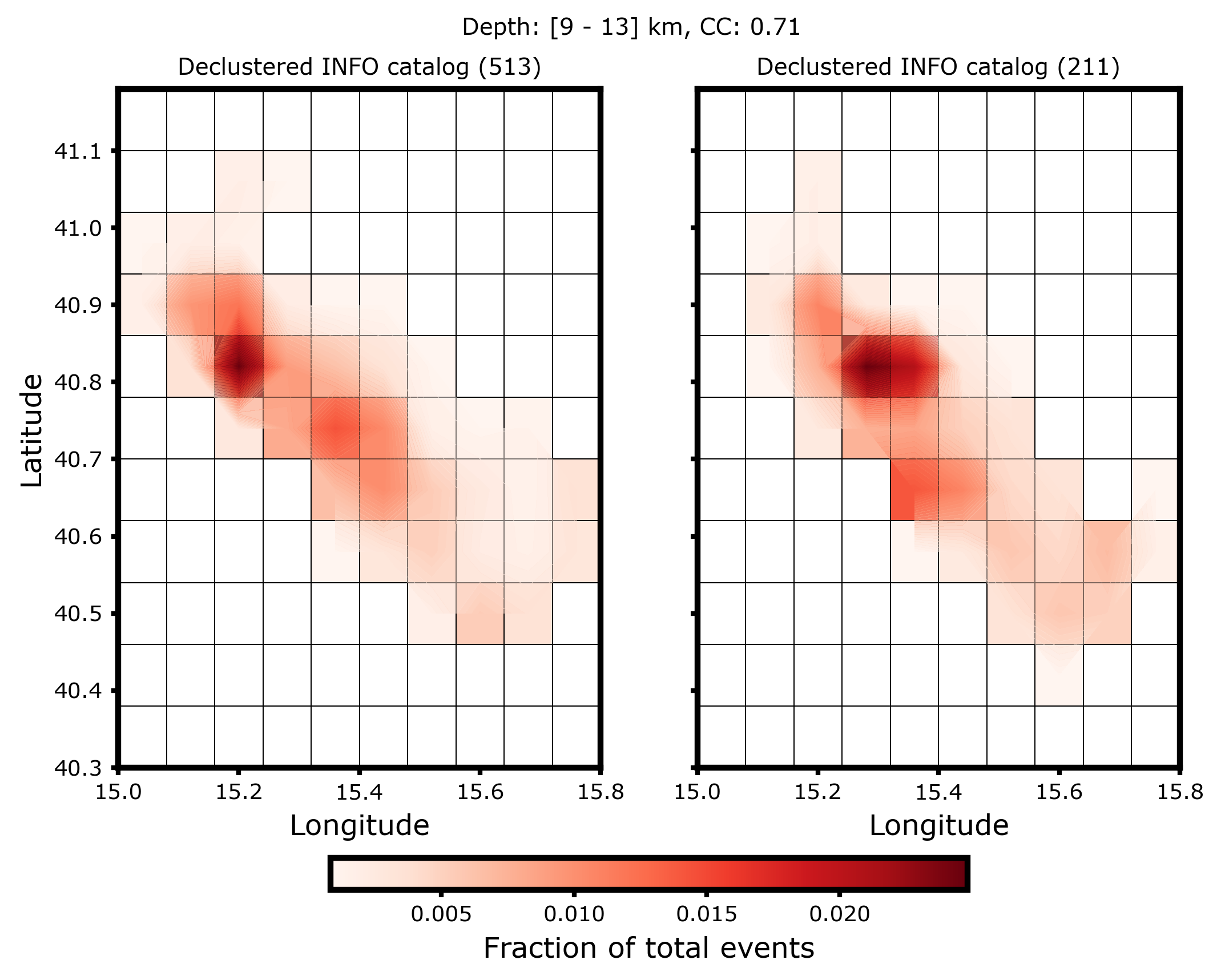

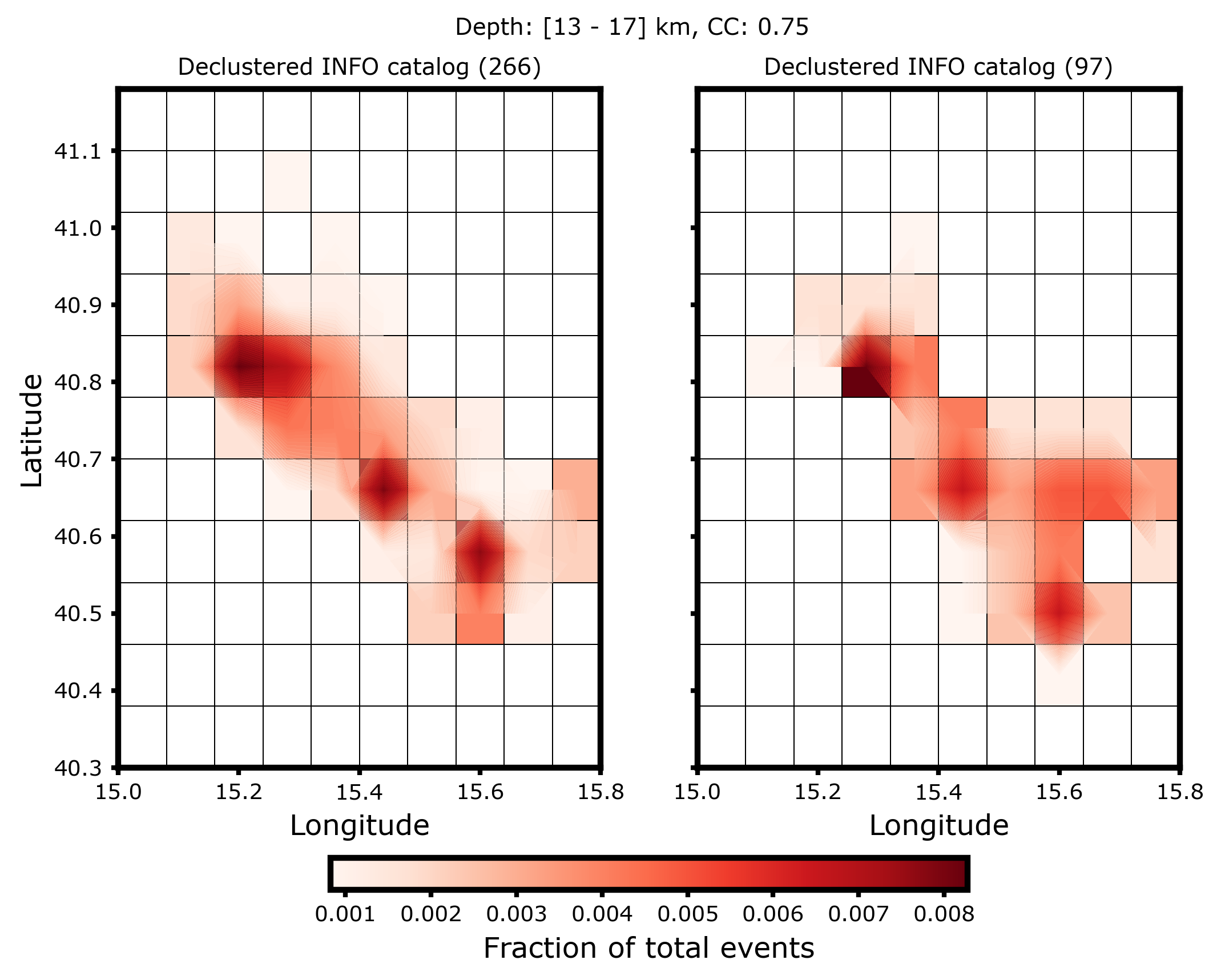


**Figure S5:** *Density distributions from the hypocenters of the short-term and long-term declustered catalogs, normalized to the total number of earthquakes of each catalog. We considered earthquakes occurring in a 75 km x 30 km-long area with major axis lying along the Apennine chain (1208 relocated earthquakes in the short-term declustered catalog, 2153 earthquakes in the declustered INFO catalog from 2007), extracting earthquakes within vertical profiles, centered at 3, 7, 11 and 15km of depth, respectively, considering the thickness of ±2 km. We measured the similarity between the density distributions of the two catalogs through the zero-lag cross-correlation coefficient.*

**Figure S6**


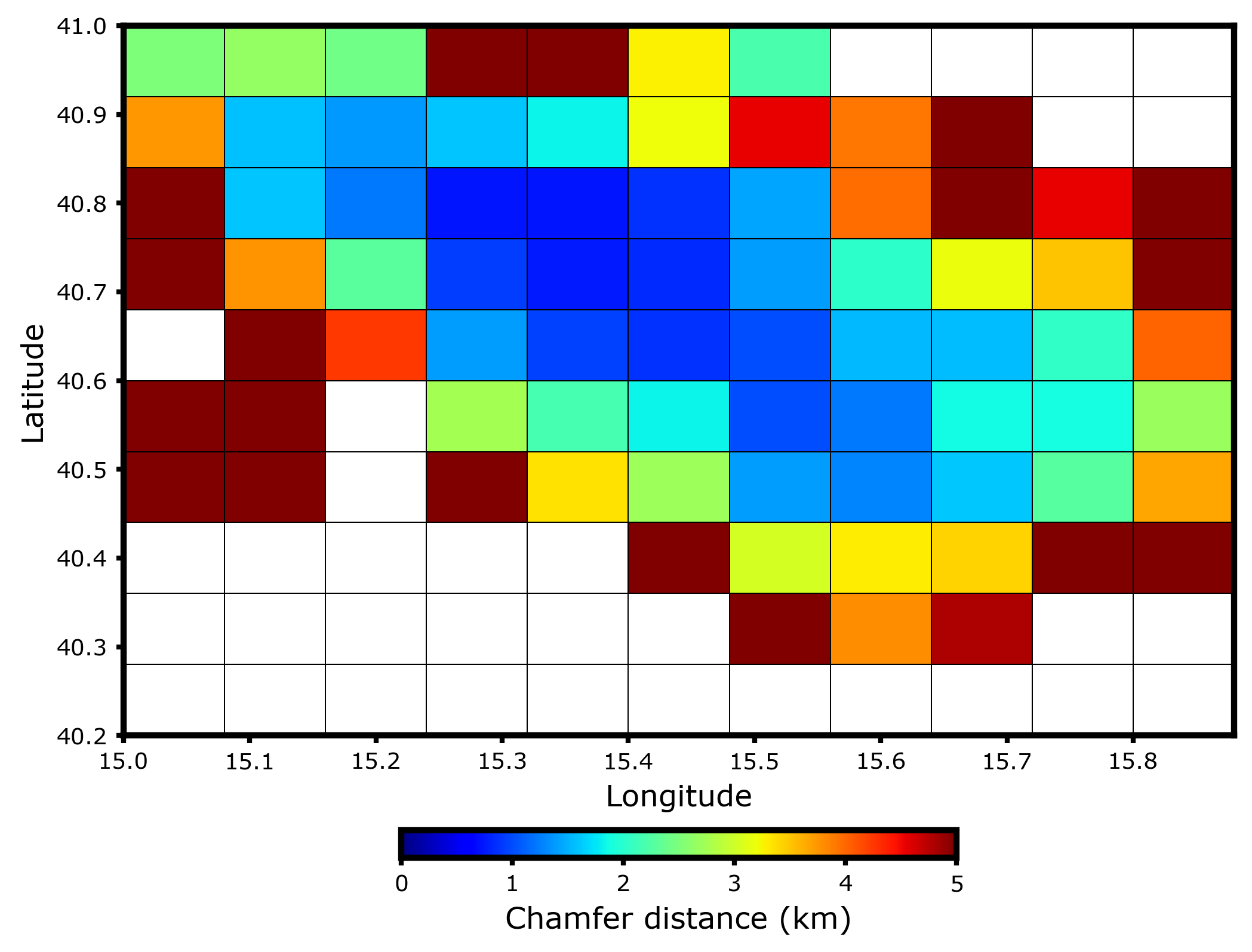


**Figure S6:** *Chamfer distance map measured between the declustered short-term DETECT and long-term ISNET within individual grid nodes as in the same grid of Figure S5.*

**Figure S7**


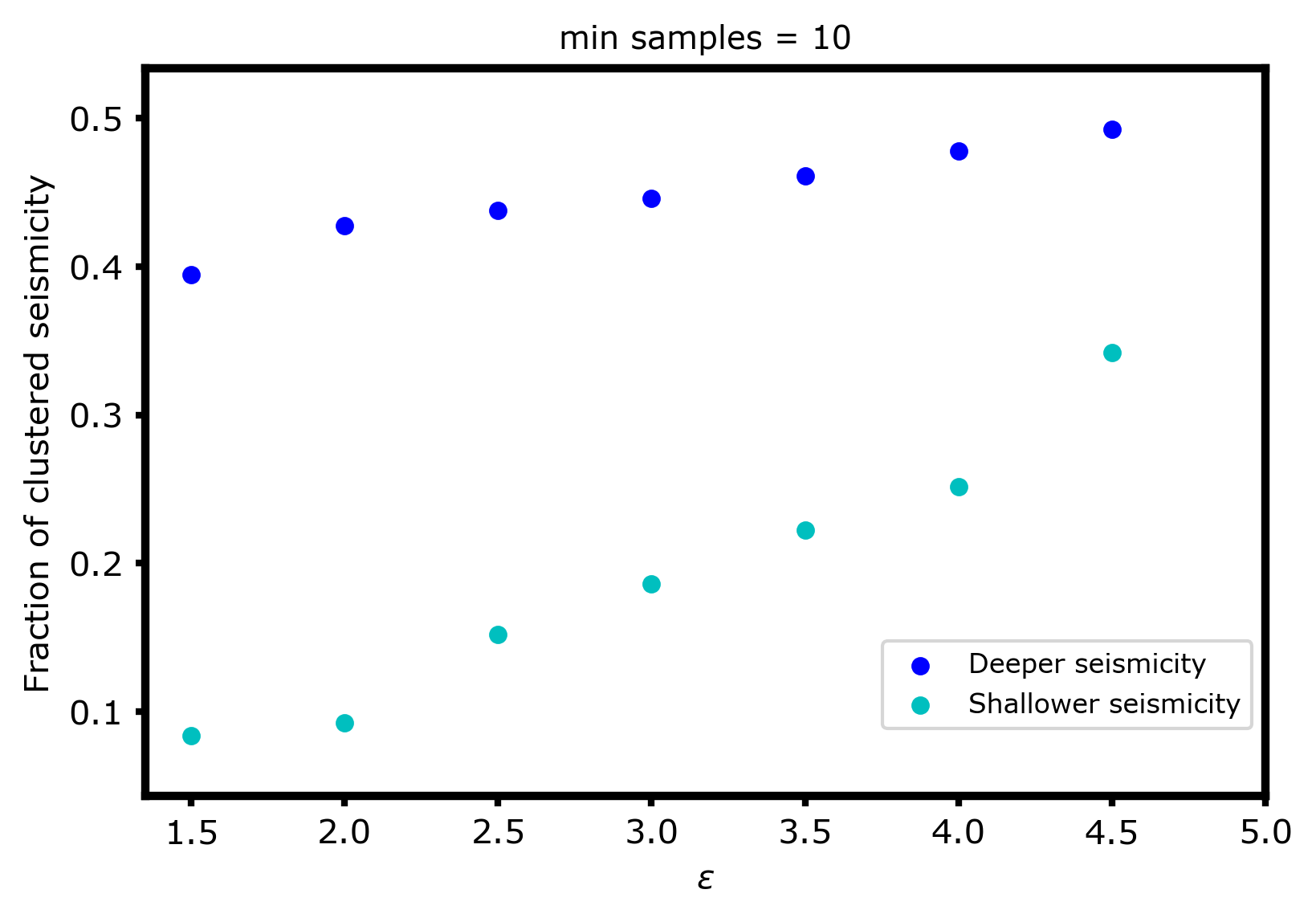

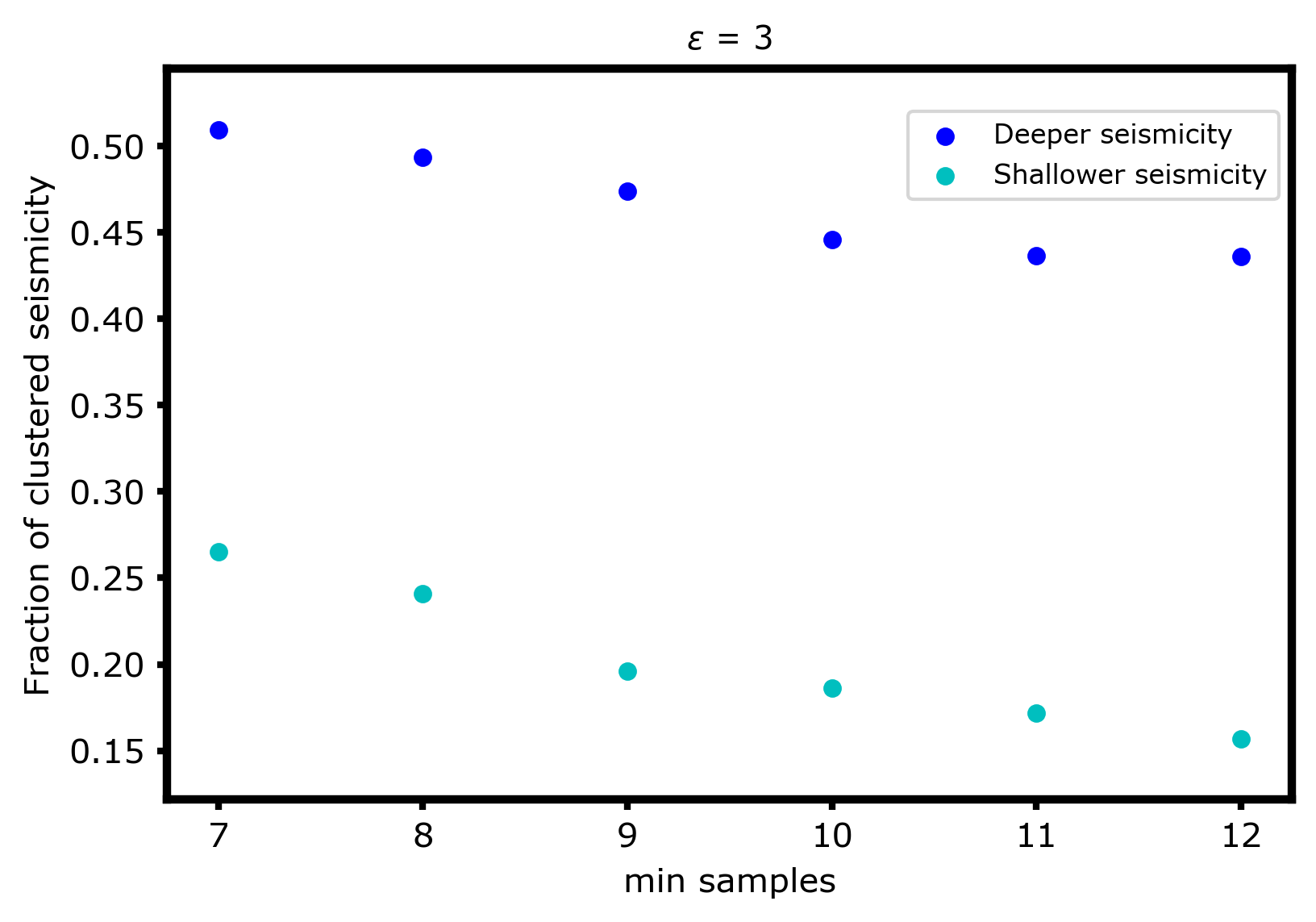


***Figure S7:*** *Tests using different parameterizations of the key DBSCAN variables, reporting the fraction of deep and shallow clustered seismicity as a function of* $\epsilon$ *and min_values. We explored* $\epsilon$ *between 1.5 and 4.5 (left panel), while ranging min_samples between 7 and 12. We observed consistent values in the fraction of clustered seismicity for events deeper than 5 km, while the fraction of clustered shallow seismicity increases as the ϵ increases (between 0.09 and 0.34).* *We retrieved more stable results when exploring variations in min_value parameter (right panel), with decreased fraction of clustered seismicity when higher values of the minimum number of neighbors are selected*

**Figure S8**


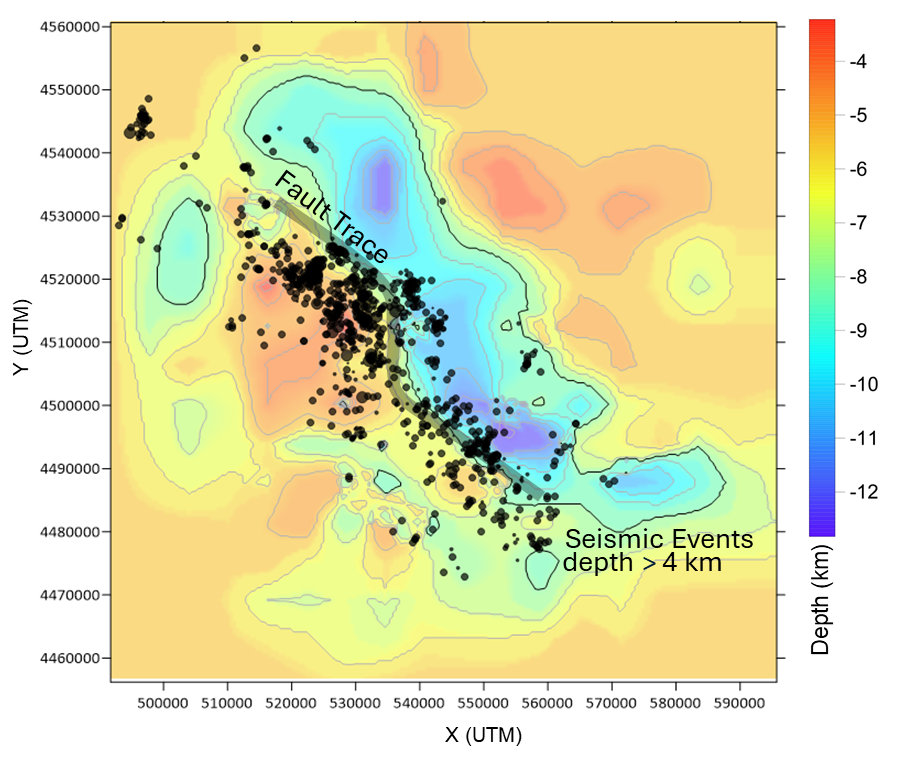


***Figure S8:*** *Isovelocity contour, colour-coded by depth, and the earthquake distribution projected onto the map view. This figure highlights the spatial consistency between the seismicity distribution from the enhanced catalog and the tomographic structure.*

**Figure S9**

| *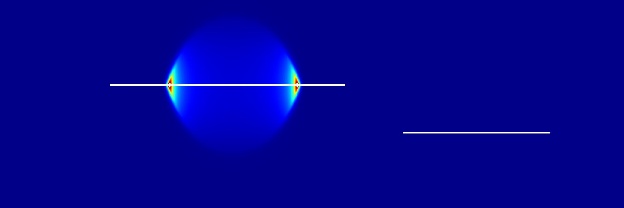* | *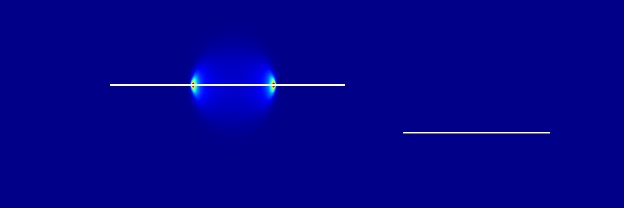* | ***t = 4s*** |
| --- | --- | --- |
| *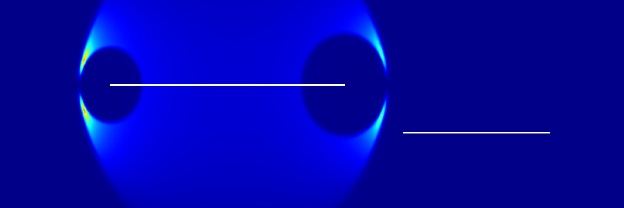* | *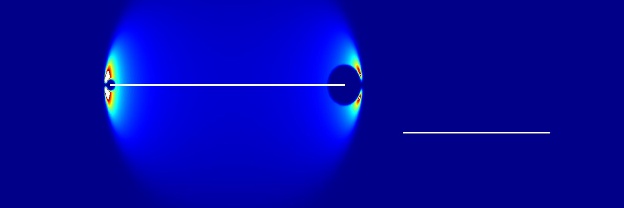* | ***t = 8s*** |
| *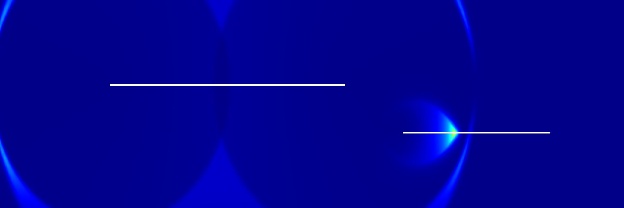* | *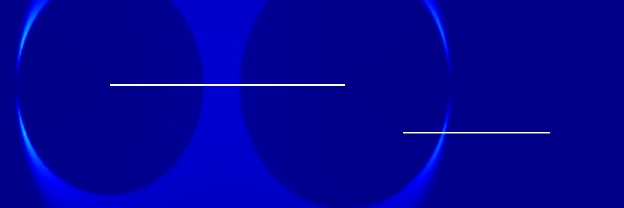* | ***t = 12s*** |

***Figure S9:*** *Numerical simulations representing scenarios that consider a step-over without a direct connection between the two segments. Most of the ruptures that develop on one segment do not jump along the other segment (right vertical panel). When the secondary segment is close to its critical state, as compared to the stress drop (the ratio between the stress excess and the stress drop referred to as the s parameter < 0.2), the rupture can nucleate on the second segment (left vertical panel)*

**Figure S10**

**
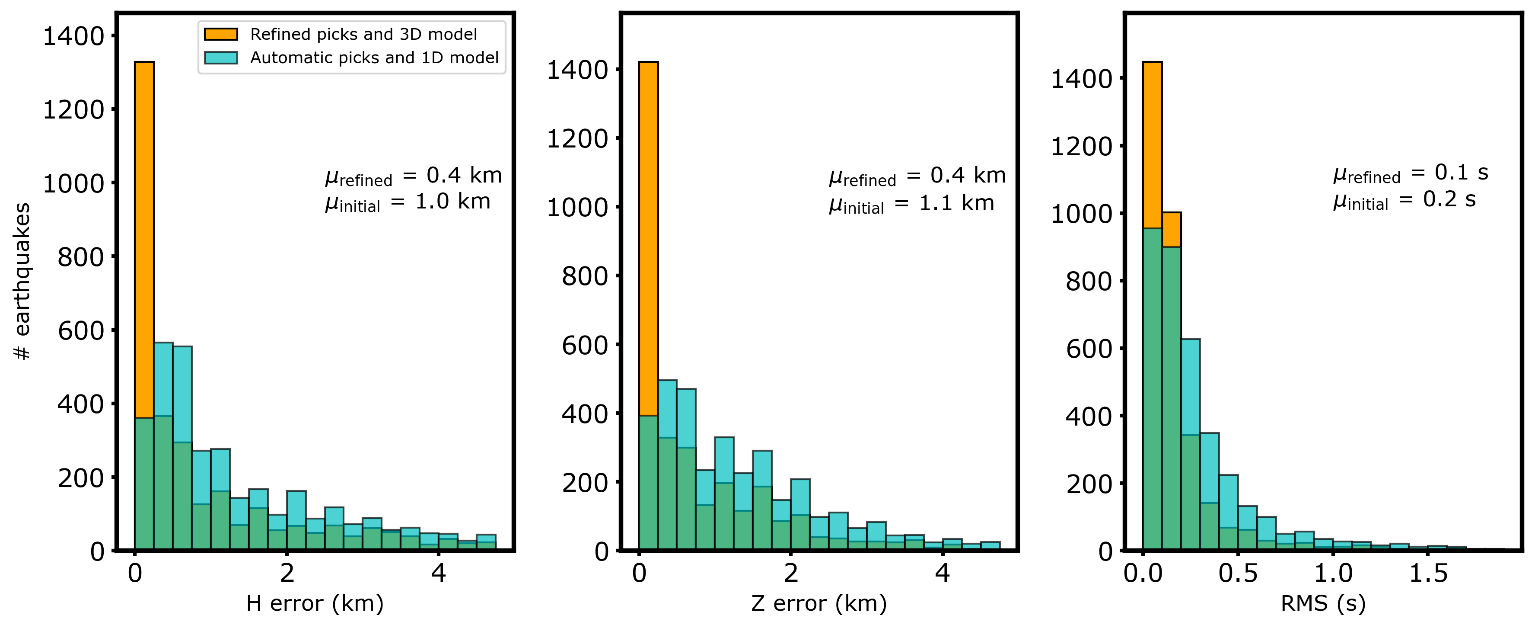
**

***Figure S10:*** *Histograms of location uncertainties (horizontal errors: left panel, vertical uncertainties: center panel, RMS of the arrival time residuals: right panel) for the absolute location from automatic phase arrival times and a 1D layer velocity model (cyan bars) and from refined arrival times and 3D velocity model (yellow bars). The implementation of refined arrival times and the adoption of a 3D velocity model can halve location uncertainties, achieving median spatial errors of 400m and RMS of 0.1s.*
